# Maternal Care in the Parasitoid *Sclerodermus harmandi* (Hymenoptera: Bethylidae)

**DOI:** 10.1371/journal.pone.0051246

**Published:** 2012-12-14

**Authors:** Zhenjie Hu, Xingli Zhao, Yisong Li, Xiaoxia Liu, Qingwen Zhang

**Affiliations:** 1 Department of Entomology, China Agricultural University, Beijing, China; 2 College of Forestry, HeNan University of Science and technology, Luoyang, China; 3 College of Agronomy, HeNan University of Science and Technology, Luoyang, China; University of Leeds, United Kingdom

## Abstract

Guarding behavior is an important activity in sub-social insects, and this behavior is believed to improve the survival of offspring. *Sclerodermus harmandi* (Hymenoptera: Bethylidae) is one of most powerful epizoic parasitoid wasps, and it parasitizes *Monochamus alternatus*, a borer of wood and also the primary vector of the pinewood nematode *Bursaphelenchus xylophilus.* After laying eggs, *S. harmandi* exhibits sub-social behavior involving the female tending the clutch of eggs until emergence (guarding behavior). In this study, the benefits of this maternal care with regard to improvements in the survival of offspring were examined. During the developmental stages, only offspring in the egg and larval stages were sensitive to guarding behavior. A positive relationship between the survival of the offspring and the duration of guarding was detected with logistic regression analysis. A female replacement experiment demonstrated that multiparous *S. harmandi* stepmothers showed guarding behavior and that this behavior improved the survival of the immature offspring, whereas nulliparous stepmothers failed to exhibit the guarding behavior. These results indicate that *S. harmandi* females display maternal care and that this behavior improves the survival of offspring.

## Introduction

Maternal care, which is typically defined as any post-ovipositional behavior that promotes the survival, growth, and development of offspring [1], is considered a hallmark of sub-sociality in insects [2]–[5]. According to the behavioral form, maternal care is classified into three groups [1], [6]: behaviors that protect offspring from natural enemies (predators, parasitoids, and microorganisms) [7], behaviors that provide/protect resources needed by the offspring [8], and behaviors that improve resources and/or facilitate resource acquisition (e.g., feeding) by offspring [9]. Among the three behavior forms, predators and parasitoids usually serve as the primary agents that lead to maternal care in insects [10]. Maternal care of offspring is age-specific and greatly improves offspring survival [11], [12]. Additionally, food provision is also considered an important form of maternal care [13]. *Forficula auricularia*, a species of earwig, provides food via mouthpart-to-mouthpart contact [14]. *Anisolabis maritime* (Dermaptera: Anisolabididae). improves the survival of its nymphs by collecting turtle food pellets even when long distances from the food resource [13].

Parental care behavior has evolved independently in at least 13 orders and at least 45 families of insects [1], [15]. Most social insect species and a relatively large family of Hemiptera display parental care [11], [16]. However, maternal care is unusual amongst parasitic wasps. Few studies have addressed the behavior of female wasps. In a bethylid wasp, *Goniozus nephantidis*, female wasps remain with offspring until pupation to protect the offspring from superparasitism, multiparasitism and conspecific intruders [17].

*Sclerodermus harmandi* (Hymenoptera: Bethylidae) is one of the most powerful epizoic parasitoid wasps against the larvae and pupae of *Monochamus alternatus* (Coleoptera: Cerambycidae), a borer of wood and also the primary vector of the pinewood nematode *Bursaphelenchus xylophilus* in Japan and China [18]. The rate of dead trees which was caused by pine nematode reduced 98.0% after release of wasps in the tested field [19]. Some studies have examined the host preferences [20], [21], development [22], biological control efficiency against different pests [23], and ultrastructure of antennae and embryos [24]–[25] of *S. harmandi*. But the adaptive significance of maternal behavior in this species is not well studied.

Here, we present our studies on the maternal care by *S. harmandi.* We addressed the following questions: (1) Was the survival of offspring improved by maternal care? (2) Which developmental stage (egg, larva, or cocoon) was sensitive to maternal care? (3) Does the guarding duration affect the survival of offspring? (4) Can replaced females, including multiparous and nulliparous stepmothers, improve the survival of the offspring?

## Results

### Behavioral observations

Upon finding that the hosts were suitable for oviposition, the female *S. harmandi* stung the host and injected venom with her ovipositor on the intersegmental membrane of the host abdomen, paralyzing the struggling host. When attacked, hosts rolled their bodies, which indicated that female were stinging the hosts and injecting venom. The female then cleaned the surface of the host to provide a habitable environment for her offspring. The female parasitoids laid a clutch of eggs onto the host surface. After a short rest, the female visited each of the eggs and patted them with its antennae gently and continuously. Moreover, the females also touched their offspring with their mouthparts and mandibles. If the eggs left the host surface, the mother clasped them with her mandibles and replaced them. During the larval stage, the female wasp bit off the epidermis in the intersegmental membrane of the hosts, resulting in some lacunae at the host surface. The larvae extended their heads into the lacunae to absorb the host haemolymph. The female parasitoids patted the offspring continuously with their antennae throughout the larval stage. When larvae overlapped, the female parasitoid clasped the overlapped larvae with its mandibles and dispersed them. If a larva died and became melanotic, the female parasitoid wasp kept it far from both the healthy larvae and the host. During the cocoon stage, the mothers still patrolled the cocoons and patted their offspring continuously. Males emerged as adults 1 or 2 days prior to females. The young male chewed a hole in the female cocoons to facilitate the emergence of the female pupae and then mated with the emerged females.

### 1. Female egg laying rate and egg mortality

**Figure 1:**
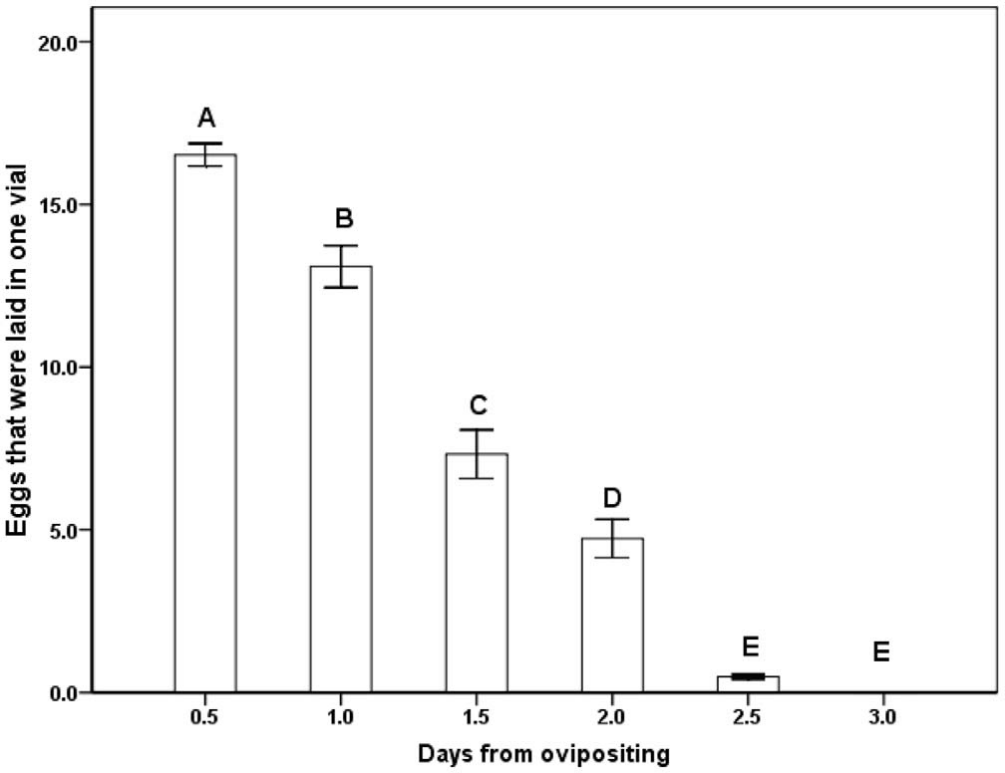
The relationship between eggs that were laid and days from oviposition. The presence of the same letter indicates a lack of significant difference (Tukey multiple comparisons test, *P*≥0.05).

**Figure 2:**
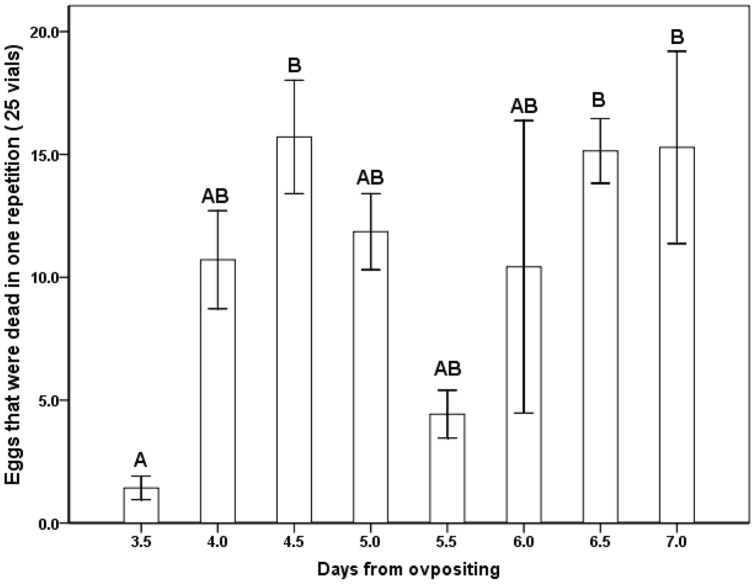
The relationship between egg that were dying and days from oviposition. The same letter at the data point indicates a lack of significant difference (Tukey multiple comparisons test, P≥0.05).

The period of laying eggs for the female *S. harmandi* was relatively concentrated in the first 2 days (Figure 1). The number of eggs laid differed significantly with days since oviposition (Figure 1. ANOVA: *F*_5_,_36_ = 186.417, P<0.001), and did not increase significantly after 2 days (Figure 1). The results showed that during the ovipositing stage, the first 2 days were the key period for female *S. harmandi* to lay eggs (figure 1). After 3 days from ovipositing, some eggs began to die (Figure 2. ANOVA: *F*_7_,_48_ = 3.367, *P* = 0.005).

### 2. Maternal care and offspring survival

Survival of offspring in the whole-stage guarding group (Table 1, group 5) was significantly higher than that in the non-guarding group (Table 1, group 1). This result indicates that the guarding behavior of female *S. harmandi* improves the survival of offspring.

**Table 1:** Survival of offspring in different groups.

Stage	Group	Mean±SE	df_1_,df_2_	*F*	*P*	95% Confidence Interval	
						Lower	Upper
ETL	1	35.41±0.88 A	6,42	38.296	<0.001	33.2594	37.5520
	2	26.19±0.61 B				24.7040	27.6845
	3	27.27±0.36 B				26.3886	28.1599
	4	35.18±0.83 A				33.1606	37.2051
	5	36.57±1.14 A				33.7641	39.3674
	6	36.02±0.99 A				33.6040	38.4303
	7	25.61±0.70 B				23.8927	27.3187
LTC	1	29.58±0.74 A	6,42	53.887	<0.001	27.7747	31.3910
	2	18.64±0.38 B				17.7088	19.5712
	3	22.33±0.35 C				21.4753	23.1876
	4	22.72±0.72 C				20.9611	24.4789
	5	29.21±0.68 A				27.5396	30.8719
	6	29.92±1.13 A				27.1502	32.6898
	7	18.78±0.47 B				17.6327	19.9330
CTA	1	24.13±0.48 A	6,42	164.273	<0.001	22.9562	25.3067
	2	12.17±0.29 B				11.4492	12.8822
	3	16.27±0.26 C				15.6387	16.8985
	4	16.67±0.36 C				15.7777	17.5594
	5	23.93±0.40 AD				22.9465	24.9164
	6	22.77±0.60 D				21.2882	24.2432
	7	11.37±0.47 B				10.2179	12.5249

ETL, egg to larval stage; LTC, larval to cocoon stage; CTA, cocoon to adult stage.

1, whole-stage guarding group; 2, non-guarding group; 3, egg-guarding hiatus group; 4, larva-guarding hiatus group; 5, cocoon-guarding hiatus group; 6, multiparous stepmother-guarding group; 7, nulliparous stepmother-guarding group. The presence of the same letter after the mean±SE indicates a lack of significant difference within group (Scheffe multiple comparisons test, *P*≥0.05).

The survival of eggs in the egg-guarding hiatus group (Table 1, ETL, group 2) was lower than that in the whole-stage-guarding group (Table 1, ETL, group 5). The survival of the larvae was lower in the larval-guarding hiatus group (Table 1, LTC, group 3) compared with the whole-stage-guarding group (Table 1, LTC, group 5). The survival rates of pupae were not significantly different between the cocoon-guarding hiatus group (Table 1, CTA, group 4) and the whole-stage-guarding group (Table 1, CTA, group 5). These results indicate that eggs and larvae were sensitive to maternal care, while cocoons were not.

### 3. The influence of guarding duration on the survival of offspring

**Figure 3:**
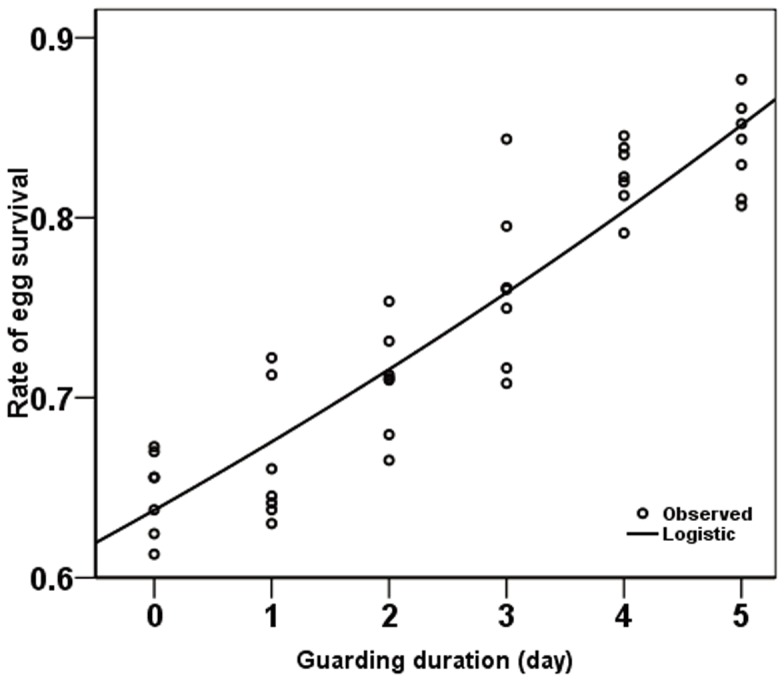
The relationship between rate of egg survival and guarding duration. Logistic regression: R^2^ = 0.842, guarding duration coefficients = 0.944, constant coefficients = 1.569; ANOVA *F*_1,40_ = 212.935, *P*<0.001.

**Figure 4:**
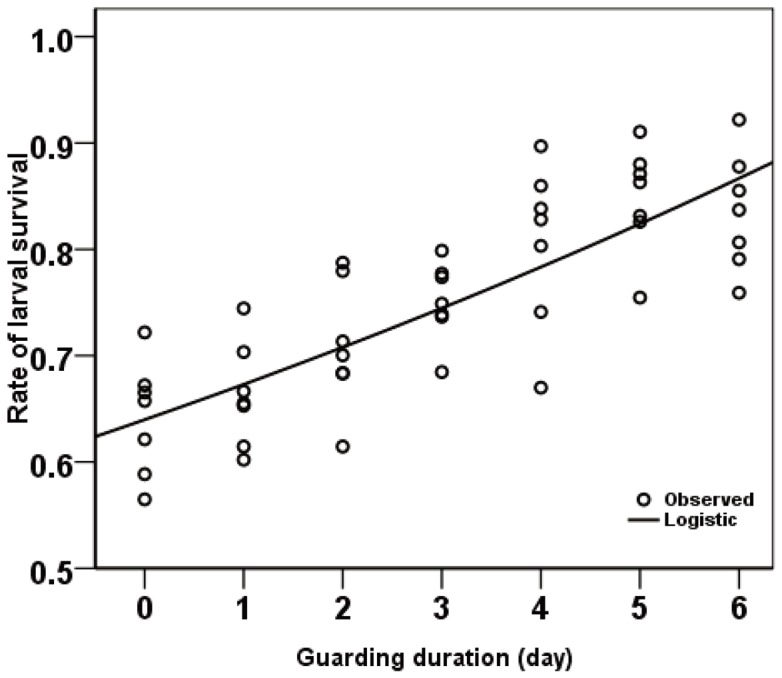
The relationship between rate of larval survival and guarding duration. Logistic regression: R^2^ = 0.656, guarding duration coefficients = 0.951, constant coefficients = 1.563; ANOVA *F*_1,47_ = 89.778, *P*<0.001.

The survival of eggs, as measured as the number of first instar larvae, was increased with increasing guarding duration. A positive correlation was detected by logistic regression between rate of egg survival and the duration of guarding behavior (Figure 3, logistic regression: R^2^ = 0.842, guarding duration coefficients = 0.944, constant coefficients = 1.569; ANOVA *F*_1,40_ = 212.935, *P*<0.001). A similar tendency was detected between the rate of larval survival and the increasing duration of guarding (Figure 4, logistic regression: R^2^ = 0.656, guarding duration coefficients = 0.951, constant coefficients = 1.563; ANOVA *F*_1,47_ = 89.778, *P*<0.001). Increased investment of time by the females resulted in higher rate of larval survival (Figure 4).

### 4. Effects of replaced females, including multiparous and nulliparous stepmothers, on survival of offspring

The survival of the offspring in the multiparous guarding group (Table 1, group 6) was higher than that in the non-guarding care group (Table 1, group 1). No difference between the multiparous-guarding group (Table 1, group 6) and the whole-stage-guarding group (Table 1, group 5) was found. This result indicates that the guarding behavior of multiparous females, like maternal females, improves the survival of offspring. The survival of the offspring was not significantly different between the nulliparous-guarding group (Table 1, group 7) and the non-guarding group (Table 1, group 1), indicating that nulliparous female rarely guarded or destroyed the conspecific offspring.

### 5. The influence of maternal care on sex ratio

Though the number of females and males was significantly different between the whole-stage-guarding group and the non-guarding group (Table 2), the sex ratio (females/[males+females]) was not significantly different between the two groups (Table 2). In conclusion, maternal care had no influence on sex ratio, and *S. harmandi* broods were female-biased.

**Table 2:** Sex differentiation in whole-stage-guarding group and non-guarding group.

	Replicates	Numbers of females	Numbers of males	Sex ratio
whole-stage-guarding group	7×25	22.88±0.450	1.20±0.092	95.02%
non-guarding group	7×25	11.55±0.286	0.62±0.033	94.91%
*t* or *χ^2^*		*t* = 21.08	*t* = 5.96	*χ^2^* = 0.205
*P*_2-tailed_		<0.001	<0.001	0.651

The numbers of females and numbers of males refer to mean±SE. The sex ratio refers to the proportion of the females (females/[females+males]).

## Discussion

Maternal care in insects is considered a hallmark of sub-sociality [1], [26], and an understanding of the evolution of maternal care offers insight into the origin of eusociality in insects [27]–[28]. The evolution of maternal care depends on the benefit of care, in terms of offspring survival, as well as the cost of future female fecundity under given ecological conditions. Maternal care only evolves when the benefits outweigh the costs of maternal care [17], [27], [29]. In this study, the benefits of maternal care in the parasitoid wasp *S. harmandi* were demonstrated as increased survival and a positive relationship between the survival of the eggs/larvae and the guarding duration. This study is, as far as we know, the first reported research about the behavior of maternal care in this species.

The forms of maternal care are diverse in insects and include nesting behavior, as well as protection and/or food provision for the whole brood and/or to individual offspring [1]–[2], [13], [16]–[17], [28], [30]. *Forficula auricularia*,a species of European earwig, guards its eggs in soil burrows throughout winter. During egg guarding, mothers continuously clean the eggs with their mouthparts to protect the eggs from pathogens [5], [31]–[33]. In a species of shield bug, *Parastrachia japonensis* (Hemiptera: Cydnidae), maternal care by provisioning increases nymphal survival under high-predation pressure environments [10], [34]. Female *Anisolabis maritima* (Dermaptera: Anisolabididae) bring food to the nest after their eggs hatch, and this behavior increases the survival of the nymphs [35]. The majority (75%) of female *P. japonensis* foraged and provisioned their nests with drupes of the host plant, *Schoepfia jasminodora* (Olacacae), to feed their offspring [36]. The female treehopper, *Publilia modesta*, guards its offspring, but the primary purpose of maternal care is to attract ants (mutualists), because ant-tending increases the number of surviving nymphs to adulthood by approximately five-fold [37]. Maternal care in *Bledius spectabili*, a subsocial intertidal beetle, protects the brood from flooding and anoxia in its burrow [38].

In the current study, we found that the survival of immature offspring was increased if the mothers stayed with their offspring. According to behavioral observations, several likely reasons for the improved survival of the offspring due to female *S. harmandi* behavior emerged. First, these insects protected the food resource from deterioration. During the process of maternal care, female *S. harmandi* launched multiple attacks on the host and injected venom. Previous studies showed that venom not only paralyzes the ability of the host to struggle but also protects the haemolymph of the host from deterioration [39]. Although the active component of venom is still unknown, previous work has demonstrated that the deterioration of host was delayed 3.8 days by the stinging of female *S. harmandi* [39]. Second, the mothers facilitated food resource acquisition [9], [14]. The female wasps bit off the epidermis of the host, resulting in some lacunae in the host surface. The larvae extended their heads into the lacunae to absorb the haemolymph of the hosts. Third, the maternal care behavior resulted in avoidance of the offspring's conspecific competition [40]. During development of larvae, the immature forms of *S. harmandi* became larger, and sometimes, this growth resulted in overlap by larvae. In these circumstances, the female wasps dispersed the larvae one by one to avoid larval competition for food and space. Finally, the maternal care provided a suitable habitat for the offspring [38]. In the case of eggs that left the host surface, the female *S. harmandi* clasped these eggs with its mandibles and replaced them on the host. Additionally, females kept the dead larvae far away from other healthy larvae to insure that healthy offspring avoided contamination by the poisonous chemicals released by the decaying (dead) larvae.

The maternal care of offspring has been reported to be associated with the stages of offspring [11], [16]–[17], [41]. Generally speaking, younger offspring are more sensitive to maternal care since these stages are more vulnerable to ecological factors, such as predators or food availability [41]. For instance, females Bethylid wasps *Goniozus nephantidis* defend younger offspring more than older offspring because the risk of younger offspring being destroyed by infanticidal intruders are higher than older offspring [42]. Maternal care is classified into 3 groups in insects according to the stage of offspring: (1) staying only with the eggs [10], [43], (2) staying with eggs until the offspring reach an advanced developmental stage [8], [17], and (3) staying with eggs until emergence [5], [20]. In this study, female *S. harmandi* stayed with its offspring until the offspring completed emergence, but only eggs and larvae were sensitive to maternal care, while the survival of the cocoons was seldom influenced by maternal care. These results likely stem from the facts that the eggs and larvae were more vulnerable compared to the cocoons and that the eggs and larvae were directly exposed to the female parasitoid and thus were available for the mother to provide assistant to improve survival, while the cocoons were insulated from the mother by the fibroin shell.

In our present study, we found that (1) multiparous stepmothers of *S. harmandi* exhibited maternal care and improved the survival of offspring and (2) nulliparous stepmothers did not exhibit maternal care but had no adverse influence, such as infanticide or superparasitism, on the survival of the offspring. In the bethylid wasp *Goniozus nephantidis*, offspring that were subjected to the guarding behavior of a stepmother suffered a high mortality [17], and the same effect occurs in the lace bug *Corythucha hewitti* [11]. The stepmothers of *S. harmandi* (including both multiparous and nulliparous stepmothers) rarely killed offspring because these stepmothers were probably siblings or originated from the same recent ancestry as the biological mother, because the mating of Bethylid wasps frequently occurs between siblings from the same brood [44]. A recent study in the Bethylid wasps showed that the relatedness between adult females mediates the degree of aggression in competitive interactions, and in concrete terms, contest behaviour was less aggressive when competitors were more closely related [45]. Furthermore, the hosts of the parasitoid *S. harmandi* are abundant in natural conditions. At least 50 species of insects in 20 families have already been reported to be suitable as hosts for *S. harmandi* [46]. Infanticide and superparasitism are more likely to occur when resources are limited [47]–[50]. For instance, one of reasons for Bethylid wasps *Goniozus nephantidis* or *Laelius pedatus* performing ovicide is that the unparasitized hosts (resources) are reared [17], [51]. Therefore, female *S. harmandi* seldom attack the parasitized host and seldom kill or destroy conspecific immature forms. Finally, according to the results of the female replacement experiment described here, we can infer that oviposition is the trigger to initiate maternal care, since only the multiparous stepmothers of *S. harmandi* exhibited maternal care. This inference was demonstrated in the whitefly *Aleyrode singularis*, as only the ovipositing females of this species exhibit maternal care [6].

In conclusion, adult female bethylid wasps of the species *S. harmandis* remained with their broods from egg stage to adulthood. This behavior benefitted the survival of the offspring. Multiparous stepmothers provided maternal care that increased the survival of the offspring; however, nulliparous stepmothers did not provide such care. Such subsocial behavior has been reported only a few times in the parasitoid wasps. The costs of maternal care in the species of *S. harmandis*, such as decreased female fecundity, and the effects of maternal care on the survival of offspring in the presence of enemies, such as predators and parasitoids, warrant further examination in the future.

## Materials and Methods

### Insects

The parasitoid *S. harmandi* was reared on the pupa of *Tenebrio molitor* L. (Coleoptera: Tenebrionidae) for 50 successive generations in our laboratory. Pupae of *T. molitor* are commonly used as a substitute for the *M. alternates* host, which are difficult to collect in abundance for the mass rearing of *S. harmandi*. The pupae of *T. molitor* were stored at 0°C for 2 days prior to use in parasitoid rearing. The adults of *S. harmandi* were placed in plastic vials (10×3 cm), and a small cotton ball with 10% honey solution was placed into each vial as a food source for the adults. The females in the study were used 10 days after emergence. Insects were kept in a climatic chamber at 24±1°C with 70% relative humidity and a 16-hour light: 8-hour dark photoperiod.

Individual female *S. harmandi* were placed in 1.2×7.5-cm glass vials that were plugged by a tampon. Each vial contained one *T. molitor* pupa. Host pupae weighed 100–160 mg. Experiments were carried out at 24±1°C with 70% relative humidity and a 16-hour light∶8-hour dark photoperiod. Each experiment comprised 175 vials (7 repetitions with 25 vials per repetition).

### The relationship between the oviposition productivity and days after emerged

One female *S. harmandi* and one *T. molitor* pupa were placed into a glass vial (1.2×7.5 cm).The pre-oviposition stage for the *S. harmandi* occurred during the first 2 days, and *S. harmandi* seldom oviposits during this period. We recorded the cumulative number of eggs from the third day (beginning of ovipositing stage) after the female and hosts were put together. If eggs were wizened, we considered them dead. The number of eggs was checked every 12 hours (0.5 day) for 7 days using an anatomical lens with a cold light source.

### Effects of maternal care on the survival of the offspring

We determined the effects of maternal care on the survival of the offspring with respect to the following issues: whether maternal care improves the survival of offspring, which stages of the offspring are sensitive to maternal care, and whether replaced females, including multiparous and nulliparous stepmothers, improves the survival of the conspecific offspring. Seven groups were designed to address the questions. In the first group, mothers stayed with their broods during the all developmental stages (i.e., egg, larva, and cocoon) of the offspring (whole-stage care group). In the second group, the mothers, after ovipositing for 2 days, were removed from the vial in order to prevent guarding behavior to the offspring (non-care group). In the third group, mothers were removed from the vial in the egg stage (egg-guarding hiatus group), in order to ensure that the offspring lacked guarding behavior in the egg stage. The removed mother was stored in an empty vial and then placed back into the vial to stay with her offspring after the eggs hatched to larvae. Similarly, in the fourth and the fifth groups, the mothers were absent in the larval and cocoon stages, respectively (larva-guarding hiatus group and cocoon-guarding hiatus group). In the sixth and seventh groups, the mothers were removed from the vial and were replaced by stepmothers that stayed with the brood until the offspring emerged as an adult. Two types of stepmothers were used: stepmothers that had previously reproduced (multiparous stepmothers) and stepmothers that had not previously reproduced (nulliparous stepmothers). Both the multiparous stepmothers and the nulliparous stepmothers were of the same age as the parental mother.

Upon hatching of the offspring from a prior stage to a posterior stage, we recorded the number of the first instar larvae, cocoons, and adults (females and males) to calculate the survival of the offspring. If the adults of the offspring were all-male broods, we excluded the samples and added new samples to yield a total of 175 vials (7 repetitions with 25 vials per repetition).

### The influence of guarding duration on the survival of offspring

Offspring in the sensitive stages were subjected to maternal guarding behavior for 1, 2, 3, 4, 5, or 6 days. During this period, the mothers stayed with the offspring in the vial. For the remaining time of a sensitive stage, the mothers were removed from the vial. The removed mothers were stored in an empty vial and then placed back into the vial to remain with the offspring after hatching from a sensitive stage to a posterior stage. We determined the offspring survival (number of immature individuals in a posterior stage, for example egg survival was defined as the number of first instar larvae).

### Data analysis and statistics

Means of each repetition (7 repetitions with 25 vials per repetition) were calculated. The statistical analyses for this study were performed using the SPSS 17.0 program for Windows. One-way analysis of variance (ANOVA, [α = 0.05]), Tukey multiple comparisons test were performed to clarify the relationship between oviposition productivity and days from ovipositing (Experiment 1). Logistic regression was performed to determine the relationship between the rate of egg/larval and guarding duration (Experiment 4). Independent-sample t-tests were performed to determine whether guarding behavior influenced the numbers of female offspring or male offspring. The Chi-Square test was performed to access the sex ratio (proportion of the females). ANOVA and Tukey multiple comparisons were performed to assess the survival of the offspring in different groups (whole-stage guarding group, non-guarding group, egg/larvae/cocoon-guarding hiatus groups, multiparous stepmother guarding group, and nulliparous stepmother guarding group). ANOVA and Tukey multiple comparisons test were carried out after the normal distribution test and homogeneity of variance test.
